# Ectopic Endometrium: The Pathologist’s Perspective

**DOI:** 10.3390/ijms222010974

**Published:** 2021-10-11

**Authors:** Alessandra Camboni, Etienne Marbaix

**Affiliations:** 1 Gynecology Research Unit, Institut de Recherche Expérimentale et Clinique (IREC), Université Catholique de Louvain, 1200 Brussels, Belgium; 2 Pathology Department, Cliniques Universitaires Saint-Luc, 1200 Brussels, Belgium; etienne.marbaix@uclouvain.be; 3 Cell Biology Unit, de Duve Institute, Université Catholique de Louvain, 1200 Brussels, Belgium

**Keywords:** endometriosis, adenomyosis, pathology, histological diagnosis, pathological classification, ovarian cancer, endometrial cancer

## Abstract

Endometriosis and adenomyosis are two frequent diseases closely linked, characterized by ectopic endometrium. Despite their benign nature, endometriosis and adenomyosis impair women’s quality of life by causing pain and infertility and an increase in the incidence of gynecological malignancies has been reported. Since the first description of ectopic endometrium in 1860, different attempts have been made to describe, classify and understand the origin of these diseases. Several theories have been proposed to describe the pathogenic mechanism leading to the development of adenomyosis or endometriosis. However, all the hypotheses show some limitations in explaining all the different aspects and manifestations of these diseases. Despite the remarkable progress made over recent years, the pathogeneses of endometriosis and adenomyosis remain unclear. Moreover, because of the lack of standardized protocols and diagnostic criteria in pathology practice it is difficult to study and to classify these disorders. The goal of this review is to summarize the pathological aspects of adenomyosis and endometriosis, spanning a historical perspective to newly reported data.

## 1. Introduction

Endometrium is a highly dynamic and regenerative tissue, under the influence of hormones, that undergoes growth and regression with each menstrual cycle, a process unique to humans and higher-order primates [1]. The regenerative potential of this tissue is probably involved in the pathogenesis of benign and malignant endometrial diseases like endometriosis, adenomyosis and endometrial cancer.

Endometriosis and adenomyosis are two common, estrogen-driven, chronic and heterogeneous diseases characterized by the presence of endometrium outside the uterine mucosa. They are quite unique as, in spite of their benign nature, they share some characteristics with neoplastic tissue. Nevertheless, their prognosis remains excellent, even if they impair women’s quality of life by causing chronic pain and infertility and representing a socioeconomic burden in term of increased medical care and decreased work productivity.

Uterine adenomyosis and endometriosis affect, respectively, 20% and 7–10% of women of reproductive age [2,3] and they frequently coexist (in 30% of cases) [4]. Common symptoms include pelvic pain and infertility and, in case of adenomyosis, abnormal uterine bleeding [4]. Besides the negative effect on women’s health, the risk of malignant transformation must be taken seriously, especially in ovarian endometriosis. Although considerable progress has been made with diagnostic imaging (transvaginal ultrasound and magnetic resonance imaging [MRI]), the gold standard in the diagnosis of adenomyosis and endometriosis is still histological examination after surgery [4]. Amid their high prevalence and severity of symptoms, our understanding of the pathogenesis and etiology of these two diseases is complex and there is no drug available to treat efficiently the diseases. Surgery is the gold standard for the management of adenomyosis and endometriosis, but it implies a risk of complications and postoperative morbidity. The absence of noninvasive diagnostic tools, lack of knowledge and non-specific symptomatology cover some of the distance yet to close in explaining underdiagnosis and undertreatment of these two conditions.

The aim of this review is to summarize current understanding of ectopic endometrium from a pathologist’s perspective. We review historical and histopathological aspects of adenomyosis and endometriosis, with particular emphasis on malignant transformation, as well as current knowledge on their pathogeneses. We also discuss the need for an exhaustive and widely accepted pathological classification to provide further insight into these complex and intriguing diseases.

## 2. Historical Aspects

Adenomyosis and endometriosis were considered to be the same disease for almost a century, until the first quarter of the 20th century. The initial description of ectopic endometrial glands in the myometrium was provided by the German pathologist Karl von Rokitansky [5], who named this condition cystosarcoma adenoids uterinum. This definition reflects the ‘tumor-like’ nature of the disease and emphasizes the two histological components of lesions: mesenchymal (endometrial stroma) and epithelial (endometrial glands). Von Rokitansky also identified two other conditions of endometrial ectopia, one showing invasion of the endometrial cavity to form polyps, known as *cystosarcoma adenoids uterinum polyposum*, and the other, infiltrating the ovary, termed *ovarian cystosarcoma*.

In 1903, Meyer described a case of iatrogenic ectopic endometrial tissue after uterine ventrofixation and elaborated the theory of ‘epithelial heterotopy’, considering the disease a sort of healing process [6]. According to Meyer, adenomyomas were examples of ‘epithelial invasion of inflammatory tissue’ occurring in both embryonic and in mature epithelium.

A few years later, the surgeon Thomas Stephen Cullen provided the first systematic description of adenomyosis [7]. With other scientists, he promoted the theory of ‘mucosal invasion’ and proposed a mechanism by which the uterine mucosa invades the underlying myometrium, in some cases demonstrating a connection between the uterine cavity and ectopic endometrium that he called an ‘adenomyoma’. He also gave a morphological and clinical explanation of what is, today, known as endometriosis and adenomyosis, describing various presentations of ‘adenomyoma’ in the myometrial wall, uterine horn, subserosa and uterine ligaments [8]. He was the first to describe decidualization of the stromal compartment during pregnancy, proving the endometrial nature of ‘adenomyoma’, and proposed hysterectomy as elective treatment [9,10].

Despite the clarity of Cullen’s hypothesis, other contemporary scientists attacked the mucosal invasion theory arguing that glands in adenomyomas were of mesonephric origin in attempts to explain ‘adenomyoma’ found in the pelvis outside the uterus [10].

In the 1920s, when Sampson proposed the theory of retrograde menstruation to explain extra-uterine disease, introducing the term ‘endometriosis’ and clarifying that adenomyosis and endometriosis were different entities [11], the endometrial origin of adenomyosis became widely accepted [12]. Two years before Sampson’s publication, Frankl (1925) introduced the term ‘adenomyosis uteri’ to describe uterine mucosal invasion of the myometrium [13]. He chose the name adenomyosis instead of adenomyositis or adenometritis so as not to suggest any inflammatory origin for the disease. In his work, he made a distinction between adenomyomas and adenomyosis: in the former, glands develop independently, while in the latter, a direct connection can be established with the uterine mucosa [14].

We then had to wait until 1972 for a widely accepted histological definition of adenomyosis provided by Bird and colleagues [15], defining adenomyosis as ‘benign invasion of endometrium into the myometrium, producing a diffusely enlarged uterus which microscopically exhibits ectopic non-neoplastic, endometrial glands and stroma surrounded by the hypertrophic and hyperplastic myometrium’.

## 3. Pathological Features

Endometriosis and adenomyosis are diseases characterized by the presence of ectopic endometrium in myometrium and extra-uterine sites respectively. At histopathological level, they appear quite similar as in both cases the main feature is the presence of normal endometrial tissue composed of glandular epithelium and stromal compartment, but they differ in clinical presentation as they show different localization. In Section 3.1 and Section 3.2, we go through an extensive description of the morphological aspects of both diseases to illustrate different histopathological features and to guide pathologists to a good diagnosis.

### 3.1. Pathological Features of Adenomyosis

Ectopic endometrial implants follow different distribution patterns in the myometrium, giving rise to two main forms of the disease: focal and diffuse. Adenomyosis is described as focal when a circumscribed nodular collection is identified but considered diffuse when different groups of endometriotic glands and stroma are distributed throughout the myometrium [3].

**Figure 1:**
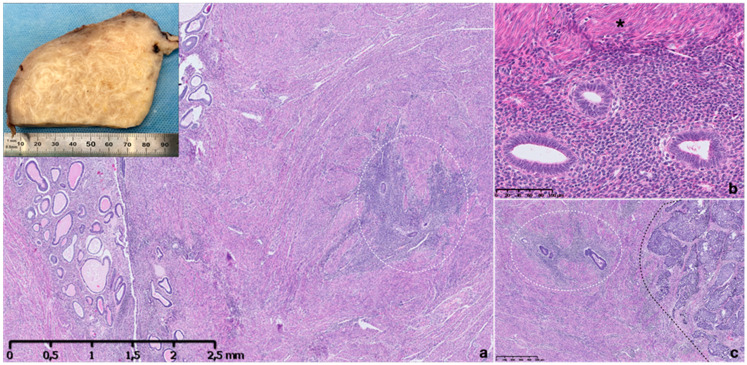
Macroscopic and microscopic appearance of adenomyosis. Thickened and trabeculated appearing myometrial wall with ill-defined hypertrophic swirls of smooth muscle of sectioned uterus with adenomyosis (**a,** inset). Histopathological image of uterine adenomyosis observed in hysterectomy specimen, with endometrial glandular and stroma invading the muscular myometrium (within circle) (**a**). Higher-power view showing ectopic endometrial glands and stroma surrounded by hyperplastic myometrium (asterisk) (**b**). Ectopic glandular epithelium is proliferative type and stroma is inactive, non-mitotic and composed of monotonous cells (**b**). A specimen showing an endometroid carcinoma infiltrating myometrial wall on the right (black line) and adenomyosis foci on the left (within line) (**c**). Hematoxylin and eosin stain.

Grossly, in the diffuse form, the uterus may look normal or atrophic in elderly women, but mostly appears asymmetrically enlarged and globular due to associated myometrial hyperplasia reflected in a thickened myometrium [16]. After cutting, the myometrium appears trabeculated, with ill-defined hypertrophic swirls of smooth muscle and petechia-like gray foci of endometrium [16] (Figure 1). Blood-filled cystic spaces may be seen, but their size does not exceed 5 mm. The focal adenomyosis, called adenomyomas, grossly mimics leiomyomas and appear as an unencapsulated mass without any well-defined border [10]. Alternatively, in some rare cases, adenomyosis may also present as a large cyst (cystic adenomyoma) [3] and occasionally assume a polypoid form and protrude into the uterine cavity mimicking an endometrial polyp.

Microscopically, histological diagnosis of adenomyosis is based on foci of endometrial stroma and glands within the myometrium, showing smooth muscle hyperplasia (Figure 1). Ectopic glands are usually inactive and resemble the basalis or proliferative-type endometrium. In about a quarter of cases, ectopic epithelium is functional and may show signs of atrophy, metaplasia or decidual change. Hemosiderin is generally absent, and glands are normally multiple and sometimes irregularly shaped. Glands in ectopic foci are not separated but interconnected [10,17], as already demonstrated in the basalis layer of eutopic endometrium that shows a different structure from those making up the functional endometrial layer [1,17]. The latter are solitary, non-branching and longitudinally arranged. This observation is intriguing and in agreement with Cullen’s vision about a connection between ectopic glands and the eutopic basal layer. Intravascular microscopic foci of adenomyosis may also be observed, raising a possible differential diagnosis with low-grade endometrial stromal sarcoma (ESS) and adenosarcoma with lympho-vascular invasion [18].

The gland-to-stroma ratio is widely variable among different foci; in some cases, the stromal component becomes undetectable and only scattered glands can be seen in the myometrium. These foci of adenomyosis with an atrophic stromal component, usually associated with typical adenomyosis elsewhere in the uterus, show atrophic glands in the absence of an infiltrative pattern or a stromal desmoplastic reaction to the glands, distinguishing this condition from invasion by a well differentiated endometrioid adenocarcinoma [19].

On the other hand, only the stromal component is observed in some rare cases. This finding is reported essentially in postmenopausal women as ‘stromal adenomyosis’ or ‘adenomyosis with sparse glands’ [20]. The complete lack of glands renders this condition difficult to diagnose and raises the question of low-grade ESS. However, the stroma in adenomyosis is inactive, non-mitotic and composed of monotonous cells, whereas, in case of ESS, lacks associated myometrial hyperplasia and shows increased stromal mitotic activity and widespread vascular involvement.

#### Pathological Classification

Adenomyosis represents a spectrum of lesions and is a progressive disease that changes in appearance during a woman’s reproductive years. The scientific community claims a widely accepted classification to facilitate communication between physicians and allow advances in knowledge of the disease.

Different histological classification systems have been proposed to correlate histological features with the presence and gravity of symptoms and provide clinical guidance for prognosis or selection of appropriate therapeutic interventions (reviewed in Table 1) [10,15,21,22,23,24,25,26,27,28,29,30,31,32,33,34,35,36,37,38,39,40,41,42,43,44,45]. Unfortunately, these attempts have not all shown reproducible results. This is related to the high variability between pathologists in the handling, sampling and analysis of specimens with adenomyosis at the macroscopic and microscopic levels. Routine histological analysis focuses on the presence or absence of adenomyosis in a limited number of samples instead of extensively mapping surgical specimens and investigation high numbers of histological sections [46]. Moreover, there is inconsistency in histopathological definitions and there are no standardized histological criteria on the depth of penetration of endometrial foci into the myometrium, so they remain arbitrary [21]. Different methods to assess glandular depth of invasion have been proposed, some using an absolute measurement and others a percentage (reviewed in Table 1). In addition, histological diagnosis of adenomyosis may depend on the quality of tissue sampling and the irregularity of the endometrial-myometrial interface [47]. To mitigate these problems, it is recommended to perform histopathological diagnosis only on well-oriented hysterectomy specimens and avoid diagnoses from curettage or hysteroscopic material.

### 3.2. Pathological Features of Endometriosis

Endometriosis may be localized inside the pelvis or in extra pelvic sites, often affecting multiple sites. Three different forms of pelvic endometriosis have been identified, namely superficial peritoneal, ovarian and deep-infiltrating endometriosis (DIE) [48]. DIE is defined as endometriosis infiltrating the peritoneum by >5 mm and is generally located in the most declivous part of the pelvis (including the pouch of Douglas, sigmoid, rectum, uterosacral and broad ligaments, vagina, ureter and bladder). In extra pelvic locations, endometriotic foci have been reported in different sites: abdominal cavity (abdominal wall, groin and perineum), abdominal organs (kidneys, liver, pancreas, bowel and biliary tract), thorax, extra pelvic nerve and muscle, skin, lymph node, nose, eyes and brain [49].

Gross pathological findings of endometriosis depend on the duration of the disease, depth of penetration of lesions, localization and timing of the menstrual cycle [50]. Endometriotic implants range from punctate foci and small stellate patches (usually less than 2 cm) to cystic, nodular or polypoid masses. Lesions show different appearances during the evolution of the disease due to accumulation of hemosiderin. They are initially unpigmented or red, then progress to mature blue or dark pigmented lesions and, finally, become white fibrotic scars [48,50,51,52]. Endometriosis appearance may change under hormonal stimulation and lesions become more swollen and congested due to bleeding during the menstrual cycle. Hemorrhages and the resulting iron accumulation will trigger inflammation leading to fibrosis, scarring and adhesions causing distortion of the normal pelvic anatomy and frozen pelvis in severe cases [53,54].

**Figure 2:**
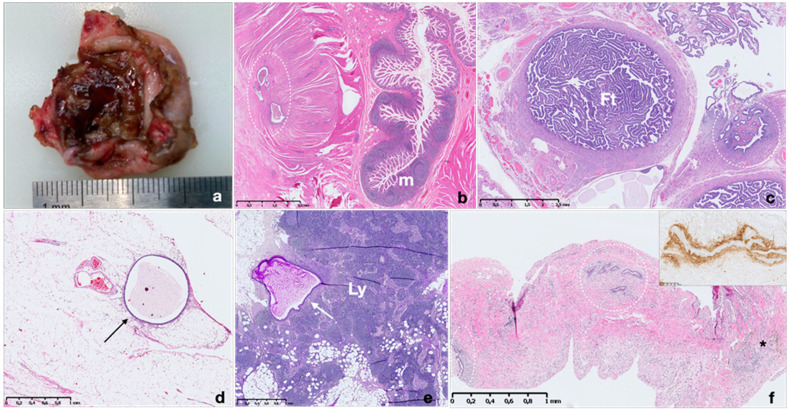
Macroscopic and microscopic appearance of endometriosis in different sites. Macroscopic picture of ovarian endometrioma with fibrotic wall and a dense, dark brown content (**a**). Histopathological images of endometriotic implants in intestinal wall, fallopian tube, mesenteric adipose tissue, lymph node and diaphragm (**b**–**f**); m: intestinal mucosa; Ft: Fallopian tube; Ly: lymph node. The withe circles and the arrows highlight endometrial implants composed by several endometrial glands and stroma (**b**–**f**). Note the presence of hemosiderin-laden macrophages (asterisk) (**f**). Immunohistochemistry for CD10 shows strong expression in stroma surrounding an ectopic endometrial gland in the diaphragm (**f** inset). Hematoxylin and eosin stain.

The most common site of involvement is the ovary, where endometriosis presents as ovarian endometriotic cysts (endometriomas) filled with blood, commonly known as ‘chocolate cysts’. In more than half of cases, endometriomas are bilateral and typically do not exceed 15 cm in diameter. Endometriomas have fibrotic walls, a smooth lining and dense, dark brown cyst contents, often adherent to adjacent organs (Figure 2).

Histological diagnosis of endometriosis is usually quite simple and is based on the presence of endometrial epithelium and stroma, or at least by one of these two elements (Figure 2).

Ectopic endometrial foci may respond to circulating hormones and may reflect the cyclic changes of eutopic endometrium, but often to a limited degree [55]. Endometriotic glands may be lined by epithelium showing an inactive to proliferative or rarely secretory appearance [50,55]. Glands are often infiltrated by pigmented histiocytes containing hemosiderin and lypofuscin as a result of hemorrhage within these foci and subsequent inflammation. Pigment may also be occasionally seen within endometriotic epithelial cells [55].

Endometriotic stroma resembles eutopic proliferative endometrial stroma and often contains network of fine vessels, which may be engorged by erythrocytes. It may undergo smooth muscle metaplasia, fibrosis, decidualization during pregnancy or progestative treatment and could be myxoid (particularly in pregnancy). In rare cases, endometriosis may lack glands, with stroma as the only identifiable component (stromal endometriosis) [50,55,56]. In these cases, evidence of chronic hemorrhage (hemosiderin-laden or foamy macrophages) may be helpful for diagnosis.

Diagnosing endometriosis may be challenging when the glandular or stromal compartment is altered or absent, in case of small-scale sampling, or when it involves unusual sites and when showing features occasionally resembling neoplasms (necrotic pseudoxanthomatous nodules, vascular, lymphatic and perineural infiltration) [55,56]. In some of these instances, immunohistochemistry may be useful to determine the diagnosis. Like normal and neoplastic endometrial stromal cells, endometriotic stromal cells are typically immunolabelled by antibodies against the CD10 antigen (also known as neprilysin, membrane metallopeptidase, neutral endopeptidase, common acute lymphoblastic leukemia antigen (CALLA)) [57,58]. CD10 is a useful immunohistochemical marker and can help confirm the endometriotic nature of stromal cells in challenging cases (Figure 2f inset). However, its expression may strongly decrease upon decidualization. Recently, interferon-induced transmembrane protein 1 (IFITM1) has been described as sensitive and specific marker of endometrial stroma [59,60]. IFITM1 (also known as 9–27, Leu-13 and CD225) was first characterized as part of a gene family highly inducible by type I and II interferons. The expression of IFITM1 in normal endometrium is consistently high in endometrial stroma, independent of hormonal status, hormonal therapy or menstrual cycle phase. Endometrial glands and myometrium were constantly negative for IFITM1 expression [59]. Compared with CD10, IFITM1 has demonstrated superior performance distinguishing endometrial stroma in ovarian and extragenital endometriosis [61]. To highlight epithelial compartment, PAX8 has been proposed as a useful tool in detecting extragenital endometriosis. PAX8 is a highly sensitive and specific epithelial marker for primary and metastatic Mullerian epithelial tumors. PAX8 is a nephric-lineage transcription factor for organogenesis of the thyroid gland, kidney and Mullerian systems. PAX8 specificity has been demonstrated in several cases of normal and neoplastic tissue and very recently in extragenital endometriosis [62]. The nuclear expression of PAX8 in endometroid lesions is not affected by preoperative hormonal therapy or the involving sites. In clinical practice, we suggest using an immunohistochemical panel with both stromal (CD10 and IFITM1) and epithelial markers (PAX8) to increase accuracy in diagnosis of endometrioid lesions.

**Figure 3:**
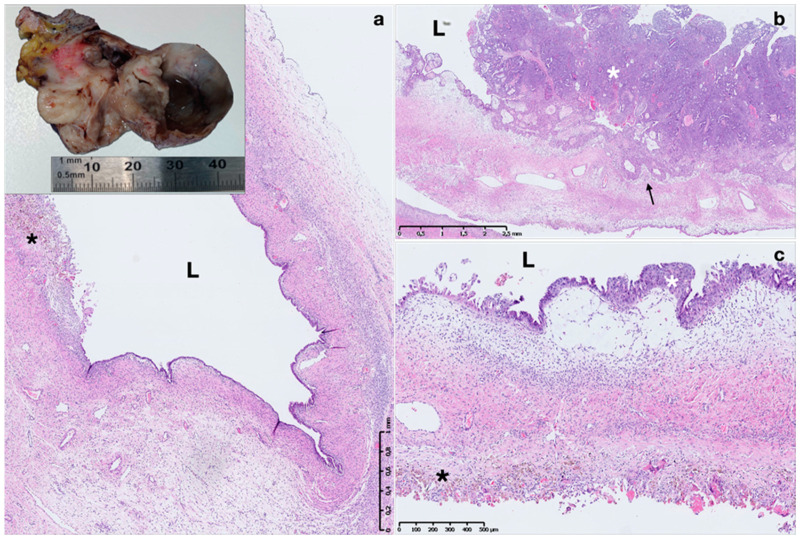
Macro- and micro-photograph of endometrioma with malignant transformation in endometroid carcinoma. A 4 cm endometrioma with cystic and solid components and a papillary lesion arising from the cyst wall (**a** inset). Histopathological image of endometriotic cyst with wide lumen lined by a single layer of columnar epithelium without atypia surrounded by relatively scant stroma (**a**). Part of the cyst wall shows the eroded lining epithelium replaced by hemosiderin-laden macrophages (dark asterisk) (**a**) and part shows a proliferation of atypical glandular cells arranged in a papillary structure (white asterisk) and invading the stroma (arrow) (**b**). Higher-power view showing an atypical lining epithelium with hobnail cells, large vesiculated nuclei with prominent nucleoli, nuclear pleomorphism (white asterisk) (**c**). L: cyst lumen. Hematoxylin and eosin stain.

Atypical endometriosis, described in 1.7–5.8% of endometriotic lesions, is characterized by cytological atypia (hobnail appearance, enlarged hyperchromatic nuclei, abundant eosinophilic cytoplasm, increased nuclear-to-cytoplasmic ratio) in the epithelial lining of glands associated or not with glandular crowding and stratification, resembling endometrial intraepithelial neoplasia (EIN) (Figure 3) [56,63]. Atypical endometriosis is considered the precursor to endometriosis-associated carcinoma (clear cell or endometrioid) and may be in continuity with these tumors [56,63].

#### Pathological Classification

Different classification systems, based on pathological findings, have emerged since the 1920s, when initial attempts were made to characterize endometriosis. In 1921, Sampson proposed a classification for ovarian endometriosis as a subgroup of hemorrhagic cysts of the ovary, proposing an etiology for the disease [22]. Wicks and Larson (1949) developed a classification system based on histological features [64] and later, Acosta (1973) developed a classification based on surgical findings [65]. In 1997, Nisolle and Donnez, identified three different forms of pelvic endometriosis based on morphologic, morphometric, and histochemical data, each probably with its own pathogenesis: peritoneal, ovarian, and deep endometriotic nodules of the rectovaginal septum, [48]. Clinical and experimental data have supported this hypothesis, and, very recently, some somatic cancer driver mutations have been identified in the epithelial compartment of deep nodular endometriotic lesions, reinforcing the idea that deep nodular endometriosis is a specific disorder [3,66]. Few years later, Chapron proposed a classification for deep infiltrative endometriosis based on the anatomical distribution of the disease dividing it into anterior and posterior compartment [67]. Interestingly, the deep infiltrating lesion of the recto-vaginal wall and of the bladder show hyperplasia of the smooth muscle layer, as in adenomyosis. In the same year, a histological classification based on the morphological aspect of glandular epithelium and on the presence/absence of glands in endometriotic implants was elaborated by Abrao and colleagues to predict the response to treatment [68].

Unfortunately, up to the present time, none of the previously mentioned pathological classification systems were widely accepted or implemented. The lack of a reproducible and well-organized classification system is a concern into the treatment of endometriosis. The poor knowledge about pathogenesis of the disease influences our diagnostic methods and the ability to design a classification scheme. For instance, there is evidence that endometriosis may have many different appearances and change over time. An ideal classification system needs to take in account these peculiar aspects of the disease and should provide information on the severity and type of endometriosis, correlate with symptoms like pain and infertility, be reproducible and easy to perform and, finally, provide information regarding the prognosis of the disease [69].

The most widely used clinical staging system for endometriosis is the 1996 revised classification of endometriosis published by the American Society for Reproductive Medicine [70]. This system relies on all three components: (i) the evaluation of endometriotic implants (ovarian or peritoneal, superficial or deep), (ii) the degree of cul-de-sac obliteration, and (iii) the evaluation of adhesions (localization, surface area involvement and appearance). According to the score, the staging system is classified into four categories: minimal, mild, moderate or severe disease. However, although this clinical classification system is clear and provides information on the extent of the disease, it does not necessarily correlate with the severity of symptoms or with histological analysis.

## 4. Association with Others Gynecological Condition and Malignant Transformation

Adenomyosis and endometriosis may be associated and coexist with other benign or malignant gynecological diseases [4]. They may give rise to malignant transformation of epithelial and/or stromal compartment. Indeed, ectopic endometrium is subject to any disease affecting orthotopic endometrium: it may exhibit hyperplasia, atypical hyperplasia/EIN, endometrial carcinoma, endometrial stromal sarcoma and carcinosarcoma [71].

A study including 472 gynecological patients, investigating the likelihood of patients with adenomyosis to develop other uterine pathologies, demonstrated an association between adenomyosis, uterine fibroids and polyps but no association with endometrial cancer [72]. On the other hand, several studies reported a high incidence of adenomyosis in patients with endometrial cancer [73,74,75,76,77] but it remains unclear whether adenomyosis affected endometrial cancer prognosis [78] (Figure 1). The correlation between adenomyosis and endometrial cancer may be related to the prevalence of adenomyosis and to a more extensive and exhaustive sampling of specimens containing carcinoma compared to non-neoplastic uteri. Endometrial cancer arising from adenomyotic lesions is quite rare, with only 78 such case reported in a recent systematic review, in which endometroid carcinoma was reported as the most common cancer subtype [46].

By contrast, endometriosis is currently classified as a potential pre-cancerous lesion by the World Health Organization (WHO) histological classification of ovarian tumors [79]. In endometriosis, development of malignant neoplasm occurs in approximately 1% of cases, 75% of which arising in ovarian endometriosis [49]. Sampson reported the first case of malignant transformation of endometriosis to ovarian carcinoma in 1925, and established criteria to identify such transformation including: (i) evidence of endometriosis near the tumor; exclusion of invasion from other sources; (ii) the demonstration of carcinoma arising from endometriosis itself; and (iii) the identification of tissue like the endometrial stroma around the neoplastic endometrial glands [80,81]. In 1953, Scott added a fourth criterion: (iv) the histological demonstration of transition between endometriosis and neoplasm [82]. Studies have confirmed histological transition from cytologically ‘‘atypical’’ endometriosis in direct continuity with tumor [83] (Figure 3). About 60–80% of cases of endometrioid endometriosis-associated ovarian cancers (EAOCs) arise in the presence of atypical endometriosis. Patients with epithelial ovarian cancer, especially endometrioid and clear cell types showed higher prevalence of endometriosis than the general population [83,84]. Genomic sequencing showed common genetic alterations, such as ARID1A, PTEN, p53, KRAS and PIK3CA gene mutations in ovarian cancers and adjacent endometriotic lesions, supporting a malignant genetic transition spectrum [83,84]. EAOC often presents at an earlier stage with lower-grade lesions than non-EAOC [85]. Endometriosis is associated with type I ovarian cancer lesions, such as low-grade serous, endometrioid, clear cell and mucinous carcinomas [84]. Endometriotic patients older than 40 years may also present an increased risk of developing endometrial cancer [86].

Although the exact cellular pathways are unknown, transformation of endometriosis toward malignancy is likely multifactorial [84].

## 5. Association between Adenomyosis and Endometriosis

Endometriosis and adenomyosis are closely linked diseases and their rate of coexistence varies according to the endometriosis phenotype involved [87,88]. In 1948, Novak and De Lima already stated that ‘one cannot resist the feeling that there is some common denominator between endometrial hyperplasia and adenomyosis, and possibly also pelvic endometriosis’ [89]. Recent publications point to a similarity in hormone levels, histopathology, growth factors and MRI results between deep endometriosis and uterine adenomyosis, suggesting that they are actually two forms of the same disease [87,90,91]. They are both associated to smooth muscle hyperplasia and share a number of features in terms of molecular alterations, including immune system dysfunction, inflammation mediated by prostaglandin–endoperoxidase synthase 2 (PTGS2), neurogenesis, vasculogenesis, and oxidative stress pathways [4,92]. Moreover, experimental studies indicate that collective cell migration and EMT are implicated in the pathogenesis of both uterine adenomyosis and deep endometriosis [3,90].

## 6. Pathogenesis

### 6.1. Endometriosis

A unifying theory on the origin of endometriosis has remained somewhat illusory, as more than one disease is probably grouped under the name of endometriosis.

Several theories of histogenesis have been proposed, including a metastatic theory wherein implants originate from uterine endometrium, and a metaplastic theory, suggesting that implants arise from tissues other than the uterus. In addition, the environment, genetic predisposition, growth factors, immunity and other mechanisms may play a role in the development of this disorder.

The most widely accepted theory is that of retrograde menstruation [93]. Initially proposed by Sampson in the 1920s, this theory reasons that during menstruation, eutopic endometrium from the uterus is thrown out via the fallopian tubes into the peritoneal cavity [80]. Endometrial cells remain viable, implant on serosal surfaces outside the uterus, establishing a blood supply and triggering inflammation upon bleeding [53]. The occurrence of retrograde menstruation has been documented in up to 90% of women exhibiting bloody peritoneal fluid during the perimenstrual period [50,90]. Endometriosis is also more frequent in women with obstructive anomalies during Müllerian duct development. Other possible ways of benign metastatic spread include lymphatic or hematogenous transport of endometrial cells to distant sites [50,94].

The metaplastic theory suggests the differentiation of peritoneal epithelium or Müllerian remnant tissue into functioning endometrial cells, perhaps by environmental factors [93]. This theory is reinforced by the occurrence of endometriosis in women with Rokitansky–Küster–Hauser syndrome (lacking functional eutopic endometrium and therefore retrograde menstruation) [95]. A more recent proposal suggests extrauterine stem/progenitor cells originating from bone marrow or endothelial progenitors differentiating into endometriotic tissue.

### 6.2. Adenomyosis

Different hypotheses on the origin of adenomyotic lesions and mechanisms involved in their evolution and progression were very recently reviewed by our group [3,96]. Two main theories were proposed, one suggesting involvement of the tissue injury and repair (TIAR) mechanism in the endometrium, leading to stromal invagination into the inner layer of the myometrium with gland invasion, in association with microenvironmental factors stimulating smooth muscle cell growth. An alternative hypothesis suggests that adenomyosis may develop, de novo, from metaplasia of displaced embryonic pluripotent Müllerian remnants or differentiation of adult stem cells [3,97].

Once the disease is initiated, invasion of the myometrium is essential for progression and establishment of adenomyosis. Abnormal wound healing in adenomyotic lesions induces loss of tight cell-cell junctions, increasing motility in epithelial cells (epithelial-mesenchymal transition [EMT]) and fibroblasts (fibroblast to myofibroblast transdifferentiation [FMT]) by overactivation of the transforming growth factor beta 1 (TGF-β1)/SMAD3 pathway.

As in endometriosis, other factors are certainly involved in the pathogenesis of adenomyosis and are still under investigation. These include hyperperistaltic myometrium, the immune system and inflammation, proliferation and apoptosis resistance, angiogenesis and genetic predisposition [96].

### 6.3. Molecular Aspects in the Pathogenesis of Endometriosis and Adenomyosis

Recent next-generation sequencing (NGS) and single-cell transcriptomic studies of eutopic endometrium, endometriosis and adenomyosis [66,98,99,100,101,102] have provided evidence on the cellular origins of adenomyosis and endometriosis suggesting that both disorders originate from normal eutopic endometrium [100,102,103]. Genetic alterations involving genes such as KRAS, PIK3CA, PPP2R1A and ARIDIA have been detected in eutopic endometrium and in endometriosis and adenomyosis lesions, suggesting that both eutopic and ectopic endometrium are clonally related [66,99,100,101]. Analyses by targeted deep sequencing of whole tissue or using laser capture micro-dissection of different cellular compartments demonstrated that all the recurrent mutations described were found to occur only in the epithelial component and not in stroma [66,98,99,100]. Moreover, targeted deep sequencing analyses of epithelial cells demonstrated recurring KRAS mutations in both adenomyosis and eutopic basalis endometrial glands [99,101]. This finding suggests that adenomyosis originates from the basalis portion of endometrial glands, harboring KRAS mutation, supporting the Cullen’s invagination theory [7,8,103].

In adenomyosis patients presenting co-occurring endometriotic lesion, multi-regional sequencing reveals identical KRAS mutations in epithelial cells of eutopic endometrium, adjacent adenomyosis and co-occurring endometriosis suggesting that adenomyosis and endometriosis are oligoclonal lesions arising from endometrial cell populations carrying a specific driver mutation [100,103].

Interestingly, in endometriotic lesion, Suda and collegues [99] described higher mutant allele frequency (MAF) of mutations on cancer-associated genes compared to normal endometrial epithelium. This finding suggests that endometrial cells harboring cancer-associated mutations, once reaching the ovarian surface by retrograde flow, may present selective advantages at this ectopic site, leading to the development of endometriotic lesion with an amplification of the clone [99,103]. The findings reinforce John Sampson’s century-old retrograde hypothesis of the origin of endometriosis at genomic level [11].

## 7. Conclusions

Adenomyosis and endometriosis are benign gynecological diseases affecting many reproductive-age women that cause pelvic pain and infertility. Despite recent progress, the pathogenetic mechanisms involved in endometriosis and adenomyosis still need to be elucidated. Further pathology research is needed to develop standardized classification system to describe and report adenomyosis and endometriosis in order to avoid over- and under-diagnosis and to provide a better understanding of their prevalence and responses to treatment.

This will be of crucial importance for research and clinical studies to offering these patients a better quality of life and even effectively curing their disease.

## Figures and Tables

**Table 1:** Histological diagnostic criteria and based classification of adenomyosis in different studies.

Reference	Diagnostic Cut-Off Point	Classification
Sampson, 1921 [22]	N/A	Group 1: Invasion from within Group 2: Invasion from without Group 3: Adenomyoma (intramyometrial)
Bensen and Sneedens, 1958 [23]	>2 LPP + muscle changes	Degree of uterine involvement: Slight Moderate Marked
Sandberg and Cohn, 1962 [24]	>2 LPF (8 mm)	N/A
Bird et al., 1972 [15]	≥1 LPF (2 mm) below the endometrium basal layer	Depth of invasion: Grade I: sub-basal lesions within one LPF Grade II: up to mid-myometrium Grade III: beyond mid-myometrium.
		Degree of involvement: Slight: 1–3 glands/LPF Moderate: 4–9 glands/LPF Marked: ≥10 glands/LPF
Owolabi and Strickler, 1977 [25]	>1 LPF	N/A
Novak and Woodruff, 1974 [26]	>1 HPF	N/A
Hendrickson and Kempson, 1980 [27]	>1/4 of total uterine wall thickness	N/A
Gompel and Silverberg, 1985 [28]	>1 MPF (×100)	N/A
Nishida et al., 1991 [29]	N/A	Type 1: Continuous from the endometrium Type 2: Continuous from the serosa
McCausland et al., 1992 [30]	≥1 mm depth	Minimal Deep
Vercellini et al., 1993 [31]	>1 LPF (4 mm)	
Siegler and Camilien, 1994 [32]	N/A	Depth of penetration from the basal layer of endometrium: grades 1–3 Degree of involvement: Mild: 1–3 islands/LPF Moderate: 4–9 islands/LPF Severe: >10 islands/LPF
		Configuration: diffuse, discrete (nodular/focal)
Vercellini et al., 1995 [33]	>0.5 LPF (2.5 mm)	N/A
Parazzini et al., 1997 [34]	>0.5 LPF (2.5 mm)	N/A
Ferenczy et al., 1998 [35]	Distance between the endomyometrial junction to the nearest adenomyotic focus should be ~25% of the myometrial thickness	
Levgur et al., 2000 [36]	≥2 mm below endomyometrial junction myometrial hyperplasia	Superficial: <40% uterine wall thickness Intermediate: 40–80% wall thickness Deep: >80% wall thickness
Zaloudek and Hendrickson, 2002 [37]	>0.5 LPF (2.5 mm)	N/A
Bergholt et al., 2001 [38]	Prevalence varied when ≥1, ≥2, or ≥3 mm from the endometrial–myometrial junction was used as a cut-off point.	
Bazot et al., 2001 [39]	>2.5 mm beyond the endometrial-myometrial junction	Depth of myometrial involvement: Grade 1: 1/3 (superficial adenomyosis) Grade 2: 2/3 Grade 3: entire myometrium (deep adenomyosis) Grading according to the number of endometrial islets: Mild: 1–3 Moderate: 4–9 Severe: ≥10
Hulka et al., 2002 [40]	>0.5 LPF (2−3 mm)	Category 1 (mild): microscopic foci or only affecting the inner 1/3 of myometrium Category 2 (focal lesions) Category 3 (severe): affecting the outer 2/3 of the myometrium
Sammour et al., 2002 [41]	≥2 mm below endomyometrial junction myometrial hyperplasia	Group A: up to 25% Group B: 26–50% Group C: 51–75% Group D: >75% of myometrial thickness
Vercellini et al., 2006 [42]	>2.5 mm from endometrial junction	Depth of myometrial involvement: Mild, 1/3 Moderate, 1/3–2/3 Severe >2/3 of uterine wall
		Grades based on degree of spread: Grade 1: 1–3 islets/LPF Grade 2: 4–10 islets/LPF Grade 3: >10 islets/LPF
		Configuration: diffuse, focal or nodular.
Kishi et al., 2012 [43]	N/A	Subtypes based on magnetic resonance imaging, surgical, and histologic findings: I: intrinsic: Inner uterine layer. II: extrinsic: outer uterine layer (normal junctional zone). III: solitary adenomyosis no connection to the junctional zone or to the serosa. IV: indeterminate
Pistofidis et al., 2014 [44]	N/A	Types based on laparoscopic and histopathologic criteria: Sclerotic Nodular Cystic
Grimbizis et al., 2014 [45]	N/A	Diffuse: disease scattered throughout the musculature. Focal: affecting a restricted area (includes adenomyoma and cystic variety) Polypoid (typical and atypical) Special (rare forms)

HPF, high-power field; MPF, medium-power field LPF, low-power field; N/A, not applicable.
